# Percutaneous Right Ventricular Assist Device for Managing Decompensated Right Heart Failure in Pulmonary Arterial Hypertension

**DOI:** 10.7759/cureus.78106

**Published:** 2025-01-27

**Authors:** Devon Kelley, Ahmed Abdelmonem, Ishan Abdullah, Watipa Makhumalo, Jalil E Ahari

**Affiliations:** 1 Internal Medicine, George Washington University School of Medicine and Health Sciences, Washington DC, USA; 2 Research, George Washington University School of Medicine and Health Sciences, Washington, USA; 3 Pulmonary and Critical Care Medicine, George Washington University School of Medicine and Health Sciences, Washington DC, USA

**Keywords:** extracorporeal membrane oxygenation (ecmo), heart failure, pulmonary artery catheter, pulmonary hypertension, right ventricular assist device (rvad)

## Abstract

Management of pulmonary arterial hypertension (PAH) with concomitant decompensated right-sided heart failure refractory to conventional pharmacotherapy presents a significant and multifaceted medical challenge. Although extracorporeal membrane oxygenation has been proven as an effective emergency intervention for unresponsive cardiopulmonary failure, the alternative use of a right ventricular assist device (RVAD) holds promise in the appropriate setting. We present a case of RVAD use in a patient with life-threatening acute PAH who was successfully bridged through critical decompensation to discharge without the need for more invasive procedures.

## Introduction

Despite the theoretical benefit of increasing cardiac output to unload the right ventricle (RV), the use of right ventricular assist devices (RVADs) in the setting of pulmonary arterial hypertension (PAH) remains relatively rare due to concerns over pulmonary edema and hemorrhage from increased pressures [1,2]. Historically, RVADs have been primarily used in conjunction with left ventricular assist devices as a short-term bridge to heart-lung transplantation [3]. When conventional medical treatments for PAH fail, extracorporeal membrane oxygenation (ECMO), whether veno-arterial (VA) or veno-venous (VV), has been the primary option. However, it carries its own risks and challenges [4]. In cases where ECMO is not viable or readily available, RVADs may offer a suitable alternative. Managing acute decompensating PAH remains challenging, with a focus on optimizing RV function and preventing multiorgan failure. Still, there is a pressing need for more data on effective treatments, particularly for patients ineligible for surgical interventions such as heart-lung transplantation or ECMO [5]. RV failure and decompensated pulmonary hypertension (PH) in the ICU demand prompt, individualized therapies targeting RV support.

Despite its potential as a bridge to transplant, the use of RVADs is hindered by the risk of functional non-reversibility in patients who have experienced prolonged RV stress. Numerous studies highlight the difficulty of weaning patients from temporary percutaneous RVADs, which are associated with increased mortality due to complications such as multiorgan failure, sepsis, and thromboembolic events linked to persistent right heart failure [6-8]. The lack of a definitive pathway for RV functional recovery post-assist device implantation adds to the hesitancy, as the long-term benefits may not outweigh the risks. Further research is needed to stratify patients whose risk profiles predict functional recovery and can give better selection guidance for device placement. In this case, percutaneous RVAD support was initiated as we believed the primary driver of RV decompensation was elevated pulmonary artery (PA) pressure. Earlier and more aggressive therapy targeting PH might have yielded a different clinical outcome. However, given the rapid decline in the patient’s condition and the presence of multi-organ failure, the decision was made to provide temporary augmentation of RV function while concurrently addressing PA pressure to the greatest extent possible. Here, we present a case of severe PAH successfully managed through critical decompensation, leading to discharge without the need for transplantation.

## Case presentation

**Figure 1:**
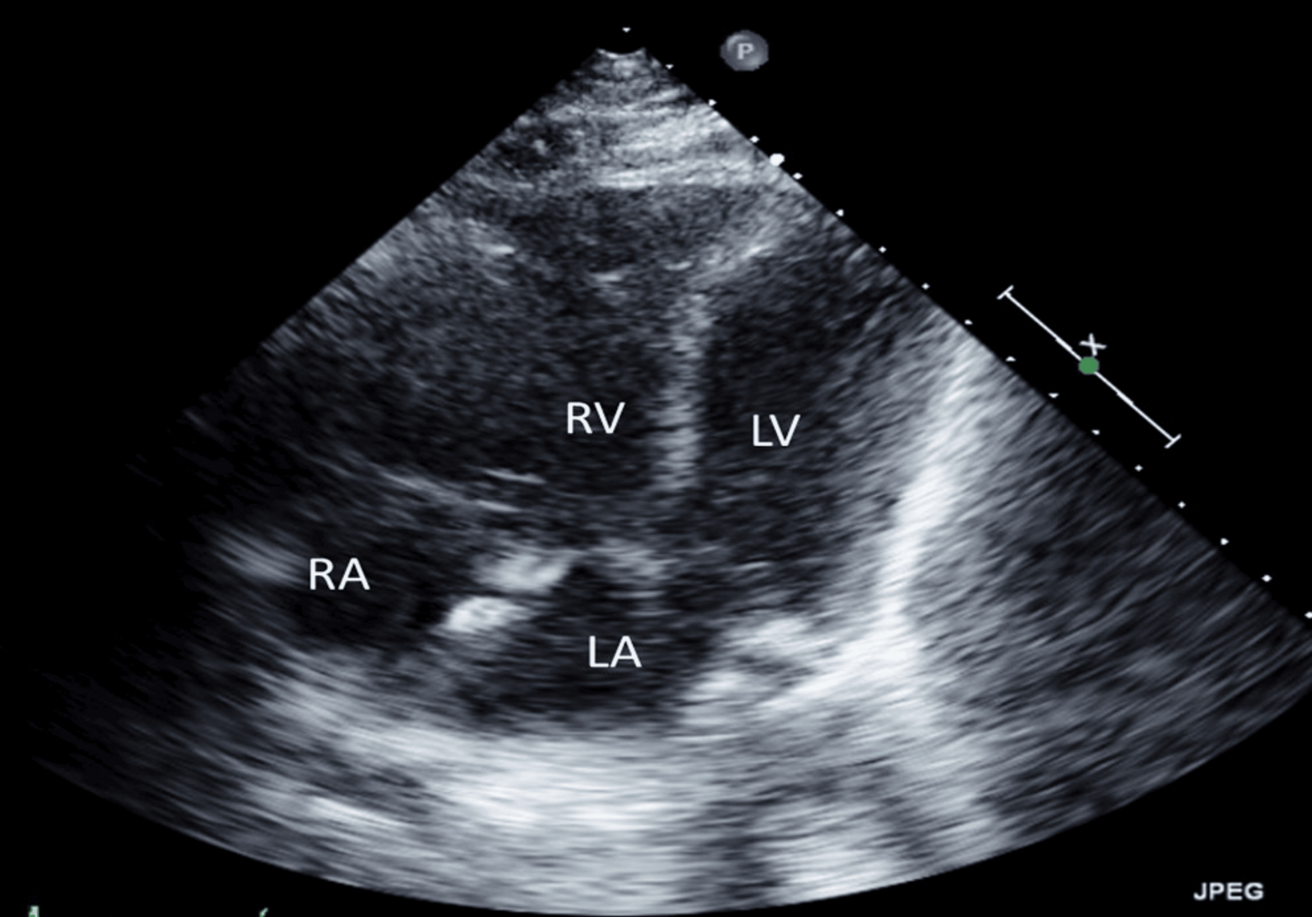
TTE showing chamber view with enlarged RV size and flattening of the septum prior to catheterization TTE: transthoracic echocardiogram, RV: right ventricle, LV: left ventricle, RA: right artery, LA: left artery

**Figure 2:**
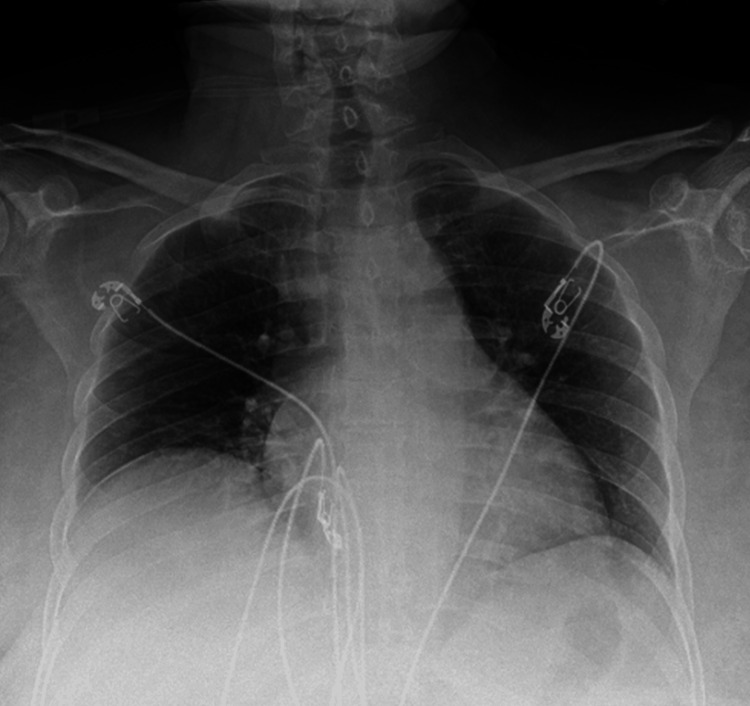
Enlarged cardiac silhouette with prominent RA shadow and absent aortopulmonary window prior to catheterization RA: right atrial

**Figure 3:**
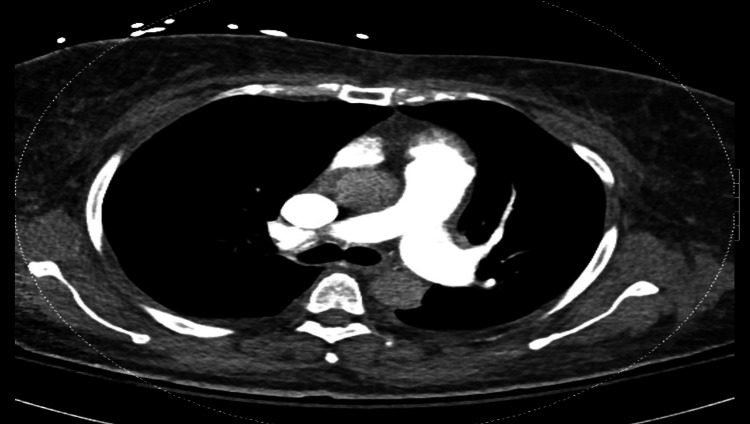
Preoperative CT showing enlarged PA CT: computed tomography, PA: pulmonary artery

**Figure 4:**
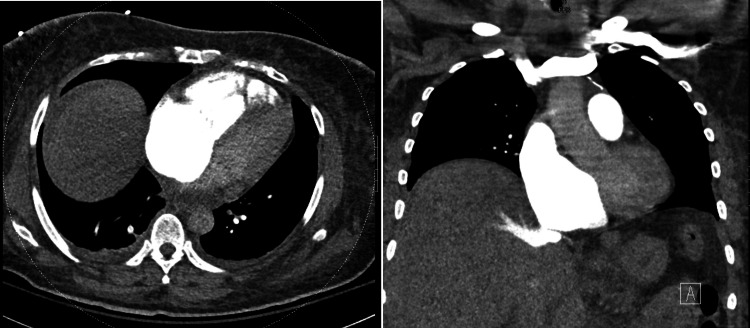
Preoperative CT showing enlarged RV CT: computed tomography, RV: right ventricle

The patient is a 59-year-old woman with no significant past medical history who initially presented to an outside hospital with complaints of shortness of breath, pleuritic chest pain, and epigastric abdominal pain. On arrival, she was noted to be tachycardic, tachypneic, and hypoxemic. Laboratory analysis demonstrated lactic acidosis at 15.8 mmol/L and mildly elevated liver enzymes ALP 121, AST 124, and ALT 113. She was transferred to our hospital for further management, where she was noted to be hemodynamically stable but with biochemical evidence of liver failure with an elevated prothrombin time and low platelets. CT angiogram scan was negative for pulmonary embolism but demonstrated contrast reflux into the liver, suggesting elevated RV pressure. A transthoracic echocardiogram further supported this, demonstrating elevated estimated PA pressures of 64 mmHg along with severely dilated RV and reduced RV systolic function with a peak RV-RA gradient of 93 mmHg (Figures 1-4).

Roughly five days after arrival at the hospital, the patient developed worsening respiratory failure, requiring transfer to the medical ICU. She subsequently developed cardiac arrest and was intubated. Following resuscitation, she was started on diuretic, inotropic, and systemic vasopressor therapy. In addition, inhaled epoprostenol was added for presumed World Health Organization (WHO) group 1 PAH, given a negative workup for PH secondary to WHO groups 2-5. She underwent placement of a Swan-Ganz catheter on day 6, which showed the following: central venous pressure of 17 mmHg, cardiac output of 3.4 L/min, cardiac index of 1.9 L/min/m2, stroke volume of 30 mL/beat, stroke volume index of 48 mL/m2/beat, stroke volume variation of 19%, baseline VO2 of 304.83, right side PA oxygen saturation of 58.8%, PA pressures of 70-80/30-40 mmHg (mean 50s mmHg), wedge pressure of 10 mmHg, and calculated pulmonary vascular resistance of 12 woods units (960 dyn.sec.cm-5).

**Figure 5:**
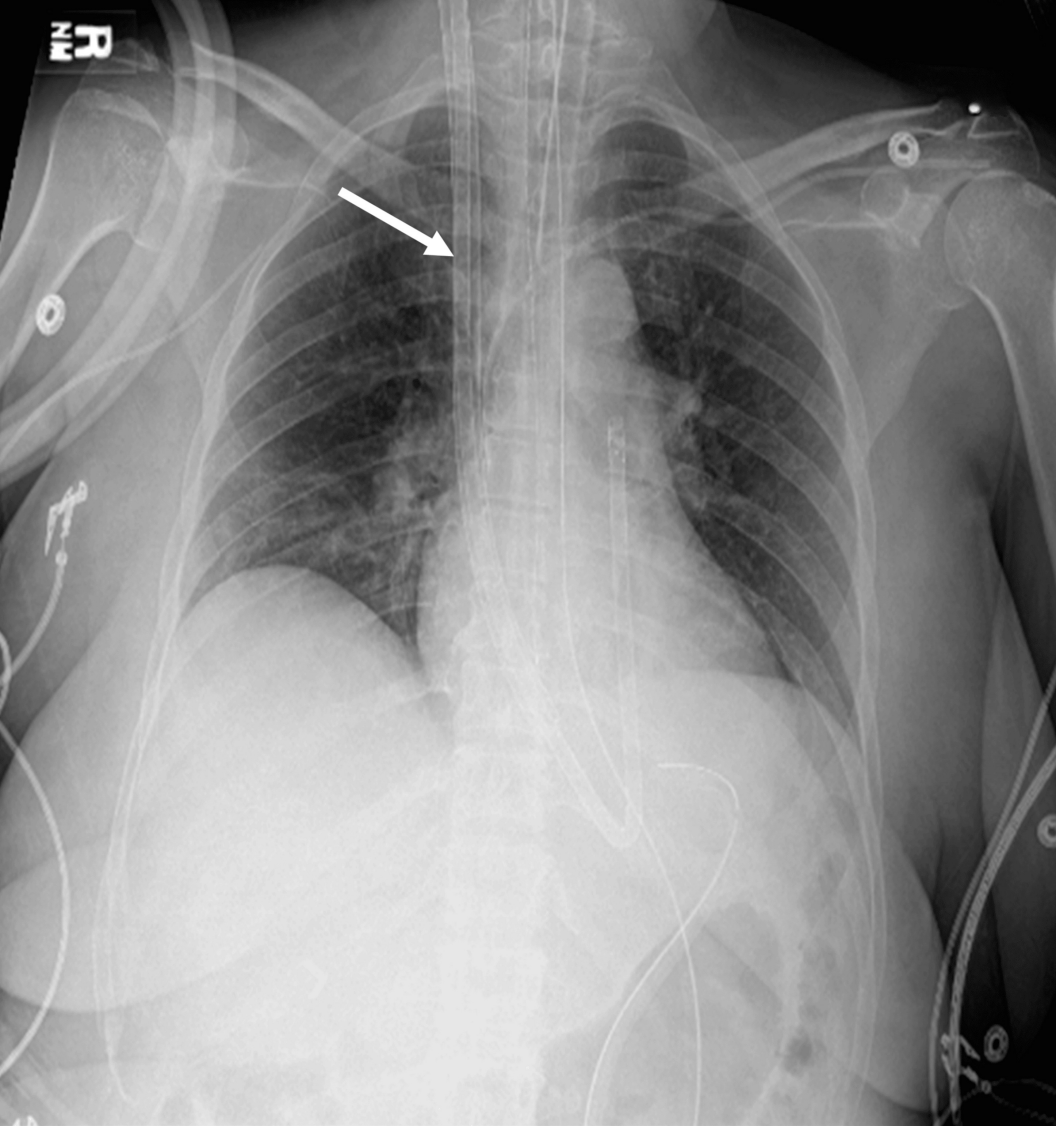
Protek Duo catheter (white arrow) with improvement showing normalized cardiac silhouette and aortopulmonary window

Escalation to intravenous (IV) epoprostenol therapy allowed for stabilization in PA pressures, and transfer to a heart and lung transplant center was initiated for a higher level of care. However, the patient began to deteriorate hemodynamically further, precluding the finalization of the transfer. As a result of rapidly progressive RV failure, she was moved to the cardiothoracic ICU. Given her tenuous hemodynamic status, as well as the rapid decline in hepatic and renal function, in addition to a lack of immediate access to ECMO, she underwent placement of a Protek Duo RVAD (TandemLife, Pittsburgh, PA, USA) (Figure 5).

Over several days, she steadily improved from a clinical standpoint, with an impressive improvement in liver and renal function tests and recovery of cardiac function coinciding with Protek Duo catheter implantation (Table 1). She experienced pulmonary hemorrhage, which was managed conservatively, leading to a tracheostomy to improve the weaning process. The pulmonary hemorrhage is believed to be due to increased RV output in the face of pulmonary vasoconstriction. The bleeding gradually stopped, the mechanical ventilator setting improved, and hemodynamic variables stabilized. IV prostacyclin therapy was slowly weaned off, and both RVAD and tracheostomy were eventually decannulated. The patient was transitioned to oral PAH-specific vasodilator therapy (sildenafil and bosentan) and discharged home on day 66 of admission with scheduled pulmonary and cardiothoracic surgery follow-up. Over time, her PAH-specific medications were changed to ambrisentan and tadalafil.

**Table 1:** Right heart catheter pressure before, during, and after Protek Duo use CVP: central venous pressure, PASBP: pulmonary artery systolic blood pressure, PADBP: pulmonary artery diastolic blood pressure, MPAP: mean pulmonary arterial pressure, PCWP: pulmonary capillary wedge pressure, CO: cardiac output, CI: cardiac index, SVO2: mixed venous oxygen saturation

	Before Protek Duo insertion	During Protek Duo insertion	After Protek Duo removal	Latest measures 6/2024
CVP	11	13	1	14
PASBP	81	84	49	69
PASBP	39	42	14	18
MPAP	52	57	49	34
PCWP	10	10	11	13
CO	2.3	4.6	7	4.5
CI	1.3	2.5	3.8	2.61
SVO2	48%	48%	73%	64%

## Discussion

The primary function of the RV is to maintain forward blood flow. To accomplish this, it must accept venous blood returning from the systemic circulation and propel it for oxygenation into a pulmonary circulation characterized by high compliance, low resistance, and low impedance. Consequently, the RV generates a fraction of the stroke work compared to the left ventricle [5]. Given this, the RV is mechanistically ill-suited for circumstances involving a sudden increase in afterload, as seen in the case of acute PAH. With inadequate treatment, the RV enters a pathological cycle in which it becomes more spherical, with increased wall tension, diastolic dysfunction, and ischemia [3,5]. Ultimately, RV failure ensues and is the predominant factor influencing morbidity and mortality in this patient population [6].

The primary therapy for PAH-induced RV dysfunction involves afterload pressure reduction. However, it is reasonable to imagine a scenario in which the RV’s ability to maintain appropriate output becomes compromised to such a degree that even lowering PA pressure does not resolve clinical instability. From a physiological standpoint, the rationale is that the RV's temporary hemodynamic offloading would supply it with the time needed for functional recovery. This can be achieved by relatively quickly and effectively decreasing RV preload, right atrial (RA) pressure, RV wall tension, and microvascular resistance, reducing RV mechanical work and oxygen demand [9].

The Protek Duo RVAD represents one of several options for mechanical circulatory support devices employed in the setting of acute RV failure [10]. Whereas many of these devices have traditionally required surgical implantation, percutaneously delivered devices, such as the Protek Duo, have emerged as viable alternatives that enable earlier intervention. The Protek Duo consists of a single double-lumen cannula. The inflow lumen is positioned in the RA and drains blood into an extracorporeal centrifugal pump. Blood is then delivered via the second lumen directly into the PA. Thus, the device serves effectively as an RV bypass system with flow capability ranging from 2-4 L/min [10,11]. Furthermore, given that venous access is obtained via the internal jugular vein, the Protek Duo has the advantage over alternative percutaneous RV support options for preserving the patient's ambulation ability [12].

Despite stabilization and improvement in PA pressures with aggressive medical management in the form of diuresis and IV vasodilator therapy, our patient’s clinical status continued to decline as a result of progressive RV failure. Her RV was in the pathological cycle described above and needed sufficient time offloaded to achieve appropriate functional recovery. The Protek Duo afforded this recovery time, with noticeable improvements in hemodynamics, hepatic, and renal function soon after placement.

One of the complications of increasing RV output in the context of PH is the risk of pulmonary hemorrhage. A recent observational study noted that prolonged RV support, specifically beyond seven days, is associated with a higher incidence of pulmonary hemorrhage [11]. Our case exhibited a similar trend, although the bleeding related to congestion was minimal and did not require intervention or escalation of mechanical ventilator support. Initially, the patient was not on anticoagulation therapy but was later started on a prophylactic dose.

A further consideration in this case, and many cases, is the availability of organs for transplant and patient waitlist status. This patient was initially too unstable to undergo lung transplantation had an organ been available to her in the emergent setting, given the acuity of her rapidly progressive right heart failure. After placing the RVAD, she remained on the device for nearly three weeks prior to decannulation, thanks to the recovery of RV function based on hemodynamic parameters. While RVAD has its pitfalls and risks, including but not limited to multiorgan system failure, thromboembolic events, and inability to wean from the device, it can provide a bridge to transplant or, in this patient’s case, a bridge to recovery. This patient remains well-managed on oral therapy alone and was able to avoid the lifelong morbidities associated with transplantation. Though it requires an extensive conversation about risks and benefits, this case demonstrates a potential use of the Protek Duo catheter RVAD as a bridge to recovery for right-sided heart failure when medical options fail, and other more advanced treatment options such as VA ECMO are not available. options have been exhausted.

Due to the relative novelty of this device and theoretical concern regarding the potential risk for pulmonary hemorrhage secondary to device-related increase in PA flow and pressure, evidence supporting the efficacy and safety of the Protek Duo is limited to a handful of case reports and case series [11-13]. Moreover, the patients in these reports for whom the device was used for PAH-induced RV failure represent only a minority, as opposed to patients with other indications (i.e., left ventricular assist device-induced RV failure). As such, we acknowledge that literature supporting the use of this device in our patient’s setting is relatively scarce (Table 2), and more data for evaluation is needed for thorough evaluation. However, this does not preclude the possibility of benefit when utilized under such circumstances, as demonstrated in our case.

**Table 2:** Previous case reports of Protek Duo catheter use in treating PH and its outcome RVAD: right ventricular assist device, RV: right ventricle, CVP: central venous pressure, VA: veno-arterial, ECMO: extracorporeal membrane oxygenation, VV: veno-venous, PA: pulmonary artery, PH: pulmonary hypertension

Author	Year	Conclusion
Vullaganti et al. [13]	2019	Temporary RVAD has improved cardiogenic shock, evidenced by decreased RV size, CVP, and improved cardiac output.
Budd et al. [14]	2019	Use of a Protek Duo cannula in a VA-ECMO configuration for hemodynamic support of a patient with severe PH and RV dysfunction undergoing lung transplant while also allowing for the seamless transition to a VV-ECMO configuration for pulmonary support in addition to RV support in an RVAD configuration.
Dyla et al. [15]	2022	Temporary RVAD support and optimal medical management may help reverse pulmonary vascular resistance, as evidenced by the improvement of PA pressure after RVAD removal.
Dolci et al., 2023 [16]	2023	Use of Protek Duo helped facilitate lung transplant by venous drainage and emptying RV.

## Conclusions

Refractory decompensation in patients with PAH and right-sided heart failure necessitates rapid intervention to prevent further complications and mortality. In settings where ECMO may be unavailable or unfavorable, alternative use of an RVAD may aid in hemodynamic stabilization and promotion of recovery. Since RV failure is a major contributor to morbidity and mortality in this setting, conventional therapy focuses on reducing afterload pressure. However, mechanical circulatory support may be necessary in cases of severe RV dysfunction to facilitate functional recovery of the RV.

This case report highlights the use of percutaneous RVAD as a temporary support strategy for RV failure secondary to PH, particularly in cases where clinicians anticipate the RV can regain its function. While discussing RVAD utilization is critical, our case underscores a pressing issue: RVAD devices are not universally available, even in hospitals capable of performing advanced cardiac interventions such as ECMO or Protek Duo catheter placement. Understanding the indications for RVAD is vital, but its accessibility is equally important, particularly when a patient’s condition is rapidly deteriorating, and timely action is crucial. The Protek Duo RVAD is a promising option for such cases, providing temporary RV offloading and facilitating hemodynamic improvement without surgical interventions. While evidence supporting its efficacy and safety remains limited, particularly in PAH-induced RV failure, the case discussed demonstrates significant clinical improvement with minimal adverse effects.
